# Breast and cervical cancer screening adherence in Jiangsu, China: An ecological perspective

**DOI:** 10.3389/fpubh.2022.967495

**Published:** 2022-08-11

**Authors:** Yanjun Sun, Yuhao Ma, Menghan Cao, Zhiqing Hu, Wei Lin, Mingsheng Chen, Yuan He

**Affiliations:** ^1^Institute of Medical Humanities, Nanjing Medical University, Nanjing, China; ^2^School of Marxism, Nanjing Medical University, Nanjing, China; ^3^School of Health Policy and Management, Nanjing Medical University, Nanjing, China; ^4^Department of Students Affairs, Nanjing Medical University, Nanjing, China; ^5^Research Center for Social Risk Management of Major Public Health Events (Key Research Base of Philosophy and Social Sciences of Universities in Jiangsu), Nanjing Medical University, Nanjing, China

**Keywords:** breast cancer screening (BCS), cervical cancer screening (CCS), ecological perspective, cancer prevention, hierarchical multiple logistic regression

## Abstract

**Background:**

High screening coverage can effectively reduce the mortality in breast and cervical cancer. Further research on extending the coverage of breast and cervical cancer screening in China is required. This study explored factors influencing women's “two-cancer” screening service utilization using an ecological approach.

**Methods:**

Data were obtained from the National Health Services Survey (NHSS) conducted in 2018 in Jiangsu, China. A total of 3,500 women aged 18–64 years were included in the analysis. Chi-squared test, hierarchical multiple logistic regression analysis, and binary logistic regression analysis were performed.

**Results:**

In total, 44.1% of the women had been screened for breast cancer (BC) and 40.9% for cervical cancer (CC). Breast cancer screening (BCS) and cervical cancer screening (CCS) differed significantly in the following common categories: age, gestational experiences, chronic disease status, body mass index (BMI), exercise, health checkup, marital status, number of children, employment, education, family doctors, and health records. In the results of hierarchical multiple logistic regression analysis, the explanatory power of the final model was 37.5% and the area under the receiver operating characteristic curve was 0.812. The results showed that being in the age group of 35–64 years, having gestational experiences, having chronic diseases, exercising, having a health checkup, being married, having children, and being employed were statistically significant positive predictors of “two-cancer” screening adherence. The household size was a barrier. For BCS, obesity was also a negative factor, and a higher overall self-related health status was a positive factor. Being married and living in households of three or more families were not predictors. For CCS, having health records was also positively significant, while having chronic disease did not influence adherence.

**Conclusion:**

The findings provide an ecological explanation for women's BCS and CCS service utilization. Both proximal and distal factors should be considered to achieve a high coverage rate.

## Introduction

Breast and cervical cancer are common malignancies worldwide. Breast cancer (BC) ranks first in incidence among female malignancies in 2020 (1). According to GLOBOCAN 2018 estimates produced by the International Agency for Research on Cancer (IARC), cervical cancer (CC) ranked 4^th^ for both incidence and mortality among all malignancies (2). The latest data from the National Cancer Center (NCC) of China showed that the crude and age-standardized incidence rates (ASIR) and age-standardized mortality rates (ASMR) of female breast and cervical cancer were both increased significantly from 2000 to 2016 (3). An upward trend in annual percentage change in screening for the “two cancers” was reported for both BC and CC. The ASIR of BC was 3% and ASMR was 1%. The ASIR of CC was 8.5% and ASMR was 5.4% (3). According to the report of IARC in 2020, the ASIR of BC, which ranked 1^st^ among the top 10 cancers with highest ASIR in China, reached 39.1 per 100,000 worldwide, and the ASIR of CC, which was 6^th^ on the same ranking, reached 10.7 (4).

Cancer screening, which is a secondary prevention, aims for early detection, diagnosis, and treatment. For breast and cervical cancer, early diagnosis and proper treatment can be life-saving. Mortality can be effectively reduced because of high coverage of cancer screening, according to the experiences of developed countries (5, 6) such as the United Kingdom and the United States (5, 7, 8). Breast cancer screening (BCS) in the United Kingdom has been nationwide as early as the 1990's. The coverage rates of cervical cancer screening (CCS) were 90% in Finland and 80% in Iceland (5). In China, a free screening program for the “two cancers” for rural women was launched in 2009 (9). In recent years, rural women's upper age limitation for participating in the program has changed from 59 to 64 years (10). The Healthy China Initiative of 2019–2030 showed that the rates of CCS and BCS are projected to reach 80% in 2022 and 90% in 2030 (11). Unfortunately, even with free screening services and encouragement from community healthcare institutions, the participation rate is relatively low, particularly in rural areas. Past research revealed that the “two-cancer” screening rate was 42.7% in Wenling, Zhejiang (12). The findings of a multistage stratified sample method in the eastern, central, and western areas of China indicated that the BSC rate in rural and urban populations was 65.6% (13). Even though the rate is gradually increasing, there could be further efforts to achieve high coverage. Therefore, to improve the status of screening service utilization, a study on what influences women's screening willingness is desired.

A previous study indicated that there are two reasons for differences in medical service utilization behavior. One reason is differences in health conditions, and the other is differences in medical services accessibility in different areas, groups, and systems (14). A previous study also showed that screening service utilization behavior is affected by multiple factors related to physical and social environments such as age, income, education, screening service delivery, perception of disease risk, and physician's recommendation (15). In this study, we will explore factors influencing women's “two-cancer” screening service utilization in China using an ecological approach that includes proximal and distal factors. The results may explain determinants of demand-side factors and supply-side factors based on women's perspectives.

## Materials and methods

### Data and sampling

Data were drawn from the 6^th^ National Health Services Survey (NHSS) collected by the National Health and Family Planning Commission (NHFPC) of China in 2018. The data used were from the province of Jiangsu. Using a multistage stratified random sampling technique, first, six districts or counties in six cities were sampled: Gusu in Suzhou, Jinhu in Huaian, Pizhou in Xuzhou, Wujin in Changzhou, Xishan in Wuxi, and Yangzhong in Zhenjiang. Then, 61 villages or resident committees were drawn from the six districts or counties. Finally, 3,660 households were selected from the village or resident committees. A total of 11,550 people were included. Given the purpose of this study, 3,500 women who were between 18 and 64 years of age and whose answers for screening, family numbers, etc., were complete were enrolled.

### Dependent and independent variables

**Figure 1:**
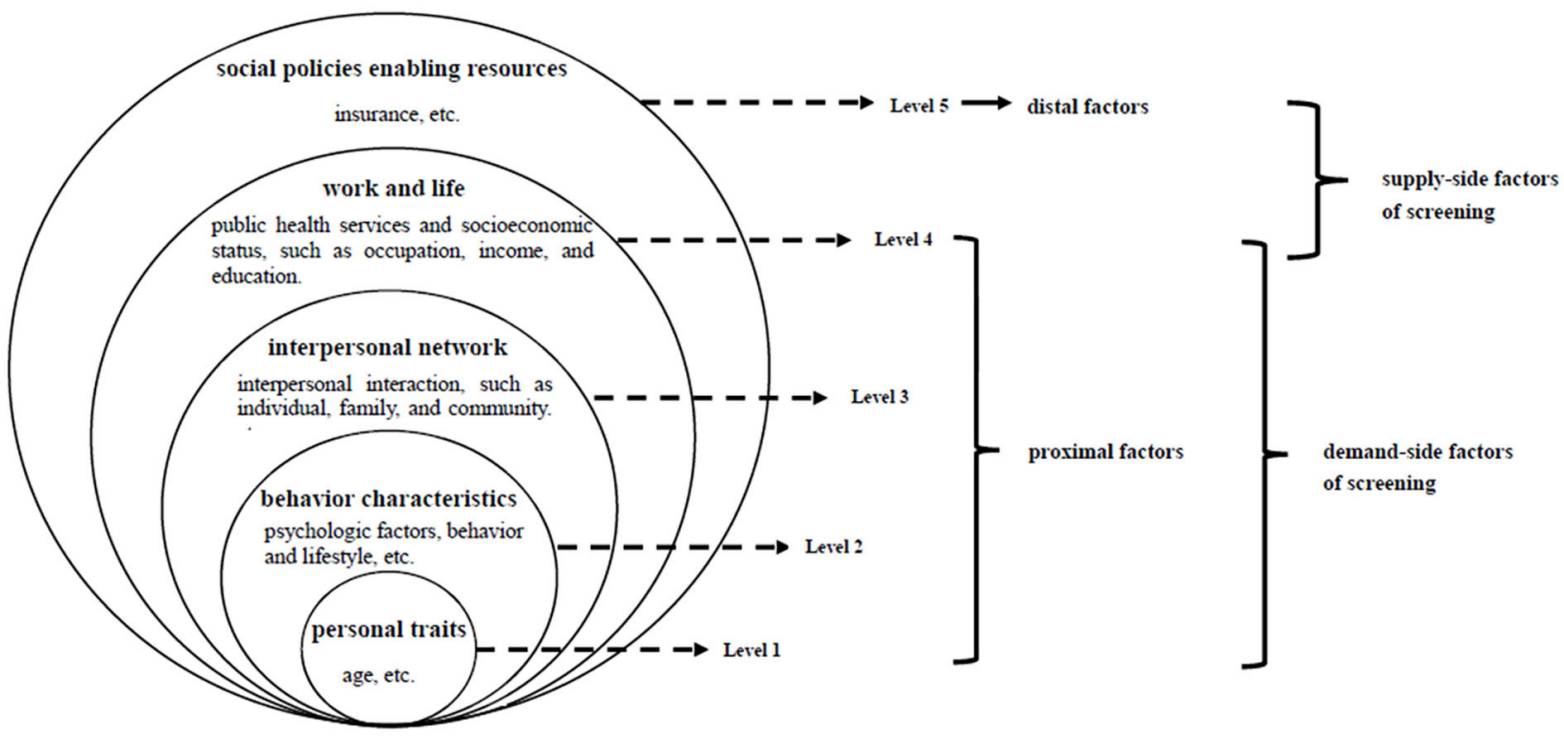
Ecological perspective on “two-cancer” screening.

The ecological perspective serves to direct attention to both behavior and its individual and environmental determinants, i.e., views of Urie Bronfenbrenner (16, 17). According to Bronfenbrenner, environmental influences on behavior are divided into the micro-, meso-, exo-, and macrosystem levels of influence. Health ecology, proposed by Collins (18), is derived from ecological theory. This is the application of ecology in the field of health. It emphasizes that an individual's health is the result of the interaction and interdependence of individual factors, health services, and both material and social environmental factors. These factors also restrict each other and affect the health of individuals and groups through multilevel interactions. According to the application of the Health Ecology Model (HEM) (Figure 1) in the health service use field (14), the determinants of health service utilization include personal traits, behavior characteristics, interpersonal network, work and life, and social policies enabling resources: (1) personal traits, the core level, refer to innate factors and predisposing characteristics of a disease, such as age, and sex; (2) behavior characteristics, the 2^nd^ level, refer to psychological factors, behavior, and lifestyle, etc.; (3) interpersonal network, the 3^rd^ level, refers to interpersonal interaction such as individual, family, and community; (4) work and life, the 4^th^ level, refer to public health services and socioeconomic status such as occupation, income, and education; (5) social policies enabling resources, the 5^th^ level, refers to insurance, etc. The first four levels are proximal factors, and the fifth level is considered a distal factor. For women's “two-cancer” screening, these levels are comprehensive and could explain the determinants of demand-side and supply-side factors based on women's perspectives (Figure 1). Combined with the measurement of the NHSS and HEM, the following dimensions (Table 1) were considered based on the existing literature.

**Table 1:** Hierarchical model of predictor variables in the study.

		**Independent variables**			**Dependent variables**
**Level 1:**	**Level 2:**	**Level 3:**	**Level 4:**	**Level 5:**	
**Personal traits**	**Behavior characteristics**	**Interpersonal network**	**Work and life**	**Social policies enabling resources**	
Age	Health behaviors	Marital status	Income	Family doctors	To be screened or not
Gestational experiences	HRQoL	Number of children	Employment	Health records	
Chronic disease status		Household size	Education	Health insurance	
BMI			Distance from the nearest hospital		

*BMI, body mass index; HRQoL, health-related quality of life*.

#### Predictor variables

The predictor variables were hierarchized into five levels. They were analyzed categorically.

#### level-1-personal-traits Level 1: Personal traits

The personal trait indices in this study included age, gestational experiences, chronic disease status, and body mass index (BMI). We calculated the prevalence of screening among two age categories according to free screening age (10) and women's average age of marriage reported in the marriage data in Jiangsu in 2017: (1) 18–34 and (2) 35–64 years. Gestational experiences were reported in three standard categories: 0, 1, or above. We assessed the presence of any chronic disease, which was defined as “with” or “without.” BMI consisted of the following four categories according to the Guidelines for Prevention and Control of Overweight and Obesity in Chinese Adults developed by the National Health Commission of the People's Republic of China in 2006 (19): (1) underweight: <18.5 kg/m^2^, (2) normal weight: 18.5–23.9 kg/m^2^, (3) overweight: 24.0–27.9 kg/m^2^, and (4) obese: ≥ 28 kg/m^2^.

#### level-2-behavior-characteristics Level 2: Behavior characteristics

Behavior characteristics were based on indicators that reflect actual health. Therefore, women's health behaviors and health-related quality of life (HRQoL) were adopted. History of smoking and alcohol consumption (in the last 12 months), health checkups (in the last 12 months), and exercise status were used to estimate health behaviors. They were all defined as “yes” or “no.” The European Quality of Life-5 Dimensions (EQ-5D) was used to estimate HRQoL including the health profile presented by the descriptive system and the overall self-rated health status presented by the EQ-5D visual analog scale (EQ VAS). Response scores on the health profile on the scale ranged from 1 (no problem) to 3 (extreme problems). Index value was calculated according to the time trade-off value set developed in China in 2018 (20). It was assessed as below or above the average. Scores on overall self-rated health status were categorized into three groups according to the level of application in the previous study of BCS and CCS (21): low (0–79), medium (80–91), or high (92–100).

#### level-3-interpersonal-network Level 3: Interpersonal network

Marital status, number of children, and household size were all included in the interpersonal network. Marital status was reported in two standard categories: single or married. Number of children was grouped as 1 or 0. Household size was grouped as 1, 2, 3 or above.

#### level-4-work-and-life Level 4: Work and life

Income, employment, education, and distance from the nearest hospital were used as indicators of work and life. Income was categorized according to median annual per capita household income. Employment status was reported in four standard categories: (1) unemployed or out of work, (2) retired, (3) employed, and (4) in-school student. Education was categorized into four groups: (1) primary school or below, (2) junior or senior high school, (3) technical school, and (4) college or above. Distance from the nearest hospital was grouped as either “ <1 km” or “1 ≥ km.”

#### level-5-social-policies-enabling-resources Level 5: Social policies enabling resources

Family doctors, health records, and insurance status were used to estimate social policies enabling resources. Family doctors and health record statuses were reported in three standard categories: “I don't know this service,” “yes,” or “no.” Insurance status was defined as “insured” or “uninsured.”

#### Outcome variables

First, the screening utilization of “two cancers” in the last 12 months was the outcome variable that was dichotomized into non-attendance in both BCS and CCS, and attendance in either one or both of them. Second, BCS attendance or not and CCS attendance or not were separately considered in order to deeply explore more specific and clearer information regarding BCS and CCS.

### Statistical analysis

A data analysis was performed using IBM SPSS Statistics version 24.0. Descriptive statistics were used on each independent variable, which was expressed in absolute value or percentage, to determine the distribution. Chi-squared tests were conducted on the following two groups: those who had attended BCS and those who had attended CCS in the last 12 months. A hierarchical multiple logistic regression analysis was conducted to evaluate determinants affecting non-attendance in both BCS and CCS, and attendance in either one or both of them in the last 12 months. Five hierarchical levels were used in this study. The independent variables were entered with the simultaneous forced entry method in the regression model by Block 1 (personal traits), Block 2 (behavior characteristics), Block 3 (interpersonal network), Block 4 (work and life), and Block 5 (social policies enabling resources). A binary logistic regression analysis was conducted to explore the potential association of the five characteristics as predictor variables and BCS attendance and CCS attendance or not as outcome variables. The standardized regression coefficient β, adjusted coefficient of determination (adjusted R^2^), and area under the receiver operating characteristic curve were observed. The results were expressed as odds ratio (OR) and respective 95% confidence intervals (CI). Differences were considered statistically significant at a two-sided *p* < 0.05. All variables integrated into the regression analysis had no missing data, so a complete case analysis was conducted.

## Results

### Characteristics of respondents

Of the 3,500 respondents, 175 (5%) were screened for BC only, 63 (1.8%) for CC only, and 1,369 (39.1%) for both BC and CC. The remaining 1,893 (54.1%) women underwent neither BCS nor CCS. The descriptive statistics for all the independent variables of the 3,500 respondents are presented in Table 2. The women's mean age was 44.66 years [standard deviation (SD) = 12.317], and 74.2% were 35–64 years of age. Of all the participants, only 8.2% had no gestation. The proportion of respondents without chronic diseases was 72.4%. Majority of the participants (59.9%) were of normal weight.

**Table 2:** Distribution of variables of participants and group differences in the different attendance groups.

**Variables**			**N (%)**	**BCS**			**CCS**		
				**No (*n*/%)**	**Yes (*n*/%)**	**χ2**	**No (*n*/%)**	**Yes (*n*/%)**	**χ2**
**Personal traits**									
Age		18–34	903 (25.8)	676 (74.9)	227 (25.1)	177.749***	712 (78.8)	191 (21.2)	196.613***
		35–64	2,597 (74.2)	1,280 (49.3)	1,317 (50.7)		1,356 (52.2)	1,241 (47.8)	
Gestational experiences		0	287 (8.2)	255 (88.9)	32 (11.1)	137.842***	266 (92.7)	21 (7.3)	146.606***
		1	991 (28.3)	522 (52.7)	469 (47.3)		566 (57.1)	425 (42.9)	
		2 or above	2,222 (63.5)	1,179 (53.1)	1,043 (46.9)		1,236 (55.6)	986 (44.4)	
Chronic disease status		Without	2,535 (72.4)	1,480 (58.4)	1,055 (41.6)	23.251**	1,552 (61.2)	983 (38.8)	17.371***
		With	965 (27.6)	476 (49.3)	489 (50.7)		516 (53.3)	449 (46.5)	
BMI		Normal weight	2,096 (59.9)	1,188 (56.7)	908(43.3)	15.830**	1,269 (60.5)	827 (39.5)	12.263**
		Underweight	220 (6.3)	142 (64.5)	78 (35.5)		143 (65.0)	77 (35.0)	
		Overweight	964 (27.5)	496 (51.5)	468 (48.5)		529 (54.9)	435 (45.1)	
		Obese	220 (6.3)	130 (59.1)	90 (40.9)		127 (57.7)	93 (42.3)	
**behavior characteristics**									
Health behaviors	History of smoking	Yes	24 (0.7)	15 (62.5)	9 (37.5)	0.429	15 (62.5)	9 (37.5)	0.117
		No	3,476 (99.3)	1,941 (55.8)	1,535 (44.2)		2,053 (59.1)	1,423 (40.9)	
	History of alcohol	Yes	202 (5.8)	99 (49.0)	103 (56.3)	4.111*	110 (54.5)	92 (45.5)	1.901
		No	3,298 (94.2)	1,857 (56.3)	1,441 (43.7)		1,958 (59.4)	1,340 (40.6)	
	The status of exercise	No	1,721 (49.2)	1,085 (63.0)	636 (37.0)	70.388***	1,126 (65.4)	595 (34.6)	56.322***
		Yes	1,779 (50.8)	871 (49.0)	908 (51.0)		942 (53.0)	837 (47.0)	
	Health checkup	No	1,643 (46.9)	1,334 (81.2)	309 (18.8)	804.457***	1,353 (82.3)	290 (17.7)	693.253***
		Yes	1,857 (53.1)	622 (33.5)	1,235 (66.5)		715 (38.5)	1,142 (61.5)	
HRQoL	Health profile	Low	692 (19.8)	380 (54.9)	312 (45.1)	0.331	391 (56.5)	301 (43.5)	2.380
		High	2,808 (80.2)	1,576 (56.1)	1,232 (43.9)		1,677 (59.7)	1,131 (40.3)	
	Overall self-rated health status	Low	800 (22.9)	463 (57.9)	337 (42.1)	2.158	476 (59.5)	324 (40.5)	2.643
		Medium	2,088 (59.7)	1,147 (54.9)	941 (45.1)		1,214 (58.1)	874 (41.9)	
		High	612 (17.5)	346 (56.5)	266 (43.5)		378 (61.8)	234 (38.2)	
**Interpersonal network**									
Marital status		Single	358 (10.2)	278 (77.7)	80 (22.3)	76.648***	294 (82.4)	64 (17.9)	87.548***
		Married	3,142 (89.8)	1,678 (53.4)	1,464 (46.6)		1,774 (56.5)	1,368 (43.5)	
Number of children		0	80 (2.3)	71 (88.8)	9 (11.3)	35.867***	75 (93.8)	5 (6.3)	40.695***
		1 or above	3,420 (97.7)	1,885 (55.1)	1,535 (44.9)		1,993 (58.3)	1,427 (41.7)	
Household size		1	110 (3.1)	54 (49.1)	56 (50.9)	2.946	58 (52.7)	52 (47.3)	2.388
		2	467 (13.3)	253 (54.2)	214 (45.8)		270 (57.8)	197 (42.2)	
		3 or above	2,923 (83.5)	1,649 (56.4)	1,274 (43.6)		1,740 (59.5)	1,183 (40.5)	
**Work and life**									
Income		Low	1,938 (55.4)	1,118 (57.7)	820 (42.3)	5.724*	1,169 (60.3)	769 (39.7)	2.736
		High	1,562 (44.6)	838 (53.6)	724 (46.4)		899 (57.6)	663 (42.4)	
Employment		Unemployed or out of work	598 (17.1)	391 (65.4)	207 (34.6)	84.484***	404 (67.6)	194 (32.4)	78.140***
		Retired	427 (12.2)	195 (45.7)	232 (54.3)		212 (49.6)	215 (50.4)	
		Employed	2,409 (68.8)	1,307 (54.3)	1,102 (45.7)		1,387 (57.6)	1,022 (42.4)	
		In-school student	66 (1.9)	63 (95.5)	3 (4.5)		65 (98.5)	1 (1.5)	
Education		Primary school or below	992 (28.3)	568 (57.3)	424 (42.7)	18.699***	579 (58.4)	413 (41.6)	26.685***
		Junior high school/ senior high school	1,580 (45.1)	824 (52.2)	756 (47.8)		877 (55.5)	703 (44.5)	
		Technical school	200 (5.7)	122 (61.0)	78 (39.0)		131 (65.5)	69 (34.5)	
		College or above	728 (20.8)	442 (60.7)	286 (39.3)		481 (66.1)	247 (33.9)	
Distance from the nearest hospital		<1 km	1,704 (48.7)	956 (56.1)	748 (43.9)	0.064	1,009 (59.2)	695 (40.8)	0.022
		≥1 km	1,796 (51.3)	1,000 (55.7)	796 (44.3)		1,059 (59.0)	737 (41.0)	
**social policies enabling resources**									
Family doctors		Don't know	2,202 (62.9)	1,300 (59.0)	902 (41.0)	27.386***	1,362 (61.9)	840 (38.1)	26.521***
		Yes	541 (15.5)	257 (47.5)	284 (52.5)		270 (49.9)	271 (50.1)	
		No	757 (21.6)	399 (52.7)	358 (47.3)		436 (57.6)	321 (42.4)	
Health records		Don't know	1,854 (53.0)	1,131 (61.0)	723 (39.0)	49.069***	1,197 (64.6)	657 (35.4)	65.804***
		Yes	1,276 (36.5)	617 (48.4)	659 (51.6)		641 (50.2)	635 (49.8)	
		No	370 (10.6)	208 (56.2)	162 (43.8)		230 (62.2)	140 (37.8)	
Insurance status		Uninsured	41 (1.2)	30 (73.2)	11 (26.8)	5.028*	30 (73.2)	11 (26.8)	3.405
		Insured	3,459 (98.8)	1,926 (55.7)	1,533 (44.3)		2,038 (58.9)	1,421 (41.1)	

*p < 0.05, **p < 0.01, ***p < 0.001. BMI, body mass index; HRQoL, health-related quality of life.

For behavior characteristics, most had no smoking (99.3%) or alcohol intake (94.2%) history. In total, 49.2% of the women never exercised or exercised less than once weekly, and 46.9% of them did not present for a routine health checkup in the last 12 months. In terms of HRQoL, the mean of EQ-5D index score was 0.9865, and 80.2% of the women scored above the average. The overall self-rated health status of more than half (59.7%) of the respondents was medium.

The majority of women (89.8%) were married. In addition, 97.7% of the respondents had one or more children. Only 3.1% of them lived alone. The proportion of women living in households with three families or more accounted for 83.5%.

The median annual per capita household income was 20,000 yuan, and 44.6% of the women had more than that. Of the 3,500 respondents, 2,409 were classified as employed and accounted for the largest proportion (68.8%), followed by being unemployed or out of work (17.1%), retired (12.2%), and students (1.9%). The number of women (45.1%) who had an educational level of junior high school or senior high school was the largest. The proportion of women whose residence was <1 km from the nearest hospital was 48.7%.

About 62.9% and 53% of the respondents reported that they did not know of family doctors and health records, respectively. Only 15.5% of the women had family doctors and 36.5% had health records. Almost all the respondents (98.8%) were insured.

### Group differences in the different attendance groups

Table 2 also presents differences between the different attendance groups according to the five variables. The BCS and CCS statuses of the women in each category are shown in Table 2. The chi-squared test results showed that going for a BCS significantly differed in all dimensions of level 1; history of alcohol (*p* < 0.05), exercise (*p* < 0.001), and health checkup (*p* < 0.001) of level 2; marital status (*p* < 0.001) and number of children (*p* < 0.001) of level 3; income (*p* < 0.05), employment (*p* < 0.001), and education (*p* < 0.001) of level 4; family doctors (*p* < 0.001), health records (*p* < 0.001), and insurance status (*p* < 0.05) of level 5. For attendance in CCS, there were significant differences in the same dimensions as BCS except for history of alcohol (*p* > 0.05), income (*p* > 0.05), and insurance status (*p* > 0.05).

### Determinants affecting attendance in “two-cancer” screening

To identify which factors influenced the screening of women's “two cancers”, a hierarchical multiple logistic regression analysis was performed (Table 3). The variable for personal traits was entered into Model 1. Even though age 35–64 years and gestational experiences were found to have significant associations with attendance in screening of the “two cancers” and this model could significantly predict women's attendance (*p* < 0.001), the explanatory power of 8.8% was not satisfactory. Variables for personal traits and behavior characteristics were entered into Model 2. In Model 2, besides age 35–64 years and gestational experiences, women who exercised every week, went for a health checkup in the last 12 months, and with a high level of overall self-rated health status were more likely to undergo screening for the “two cancers.” The explanatory power of this model increased to 36% (*p* < 0.001) compared to Model 1. Based on the significant variables in Model 2, the newly entered variables in Model 3, including marital status (married vs. single), number of children (1 or above vs. 0), and household size (2 vs. 0), were all significantly related to women's screening attendance, and had an explanatory power of 36.6% (*p* < 0.001). In Model 4, having chronic diseases and living in households of three or more families changed from not being significantly associated with screening to being predictive factors. Additionally, being employed, the newly entered variable, was also significantly associated with the outcomes. However, high overall self-rated health status was not significant. The explanatory power of this model increased to 37.4% (*p* < 0.001). In addition to the above dimensions with significant differences in Model 4, no variables were newly significant in Model 5; however, the R^2^ (37.5%) value of the final model still increased slightly (*p* < 0.001).The Hosmer-Lemeshow (H-L) test showed a good model degree of fit (*p* = 0.203). The area under the receiver operating characteristic curve was 0.812. In other words, with the entry of proximal factors and the addition of distal factors, the explanatory power increased and the model was gradually stabilized.

**Table 3:** Hierarchical multiple logistics regression analysis of factors that were related to “two-cancer” screening.

**Variables**			**Model 1**		**Model 2**		**Model 3**		**Model 4**		**Model 5**	
			**β**	**OR(95%CI)**	**β**	**OR(95%CI)**	**β**	**OR(95%CI)**	**β**	**OR(95%CI)**	**β**	**OR(95%CI)**
**Personal traits**													
Age		35–64	0.76	2.13 (1.76–2.57)***	0.79	2.20 (1.77–2.74)***	0.76	2.13 (1.71–2.66)***	0.82	2.26 (1.76–2.91)***	0.81	2.24 (1.74–2.88)***
Gestational experiences		1	1.46	4.30 (2.92–6.34) ***	1.73	5.66 (3.73–8.60)***	1.36	2.90 (2.42–6.31)***	1.28	3.61 (2.21–5.90)***	1.28	3.60 (2.20–5.89)***
		2 or above	1.34	3.82 (2.60–5.61)***	1.70	5.49 (3.62–8.31)***	1.31	3.71 (2.30–5.99)***	1.26	3.52 (2.15-5.75)***	1.25	3.50 (2.14–5.73)***
Chronic disease status		With	0.11	1.11 (0.95–1.30)	0.12	1.13 (0.93–1.38)	0.12	1.23 (0.92–1.38)	0.21	1.23 (1.003–1.50)*	0.21	1.23 (1.01–1.51)*
BMI		Underweight	0.17	1.18 (0.87–1.61)	0.20	1.22 (0.86–1.73)	0.20	1.22 (0.86–1.73)	0.23	1.26 (0.89–1.79)	0.23	1.26 (0.89–1.79)
		Overweight	0.07	1.07 (0.91–1.25)	0.01	1.01 (0.84–1.21)	−0.002	0.998 (0.83–1.20)	0.02	1.02 (0.85–1.22)	0.02	1.02 (0.85–1.23)
		Obese	−0.24	0.78 (0.60–1.04)	−0.30	0.74 (0.53–1.03)	−0.30	0.74 (0.53–1.03)	−0.28	0.76 (0.54–1.06)	−0.29	0.75 (0.54–1.05)
**Behavior characteristics**													
Health behaviors	History of smoking	No			0.54	1.71 (0.66–4.43)	0.49	1.63 (0.63–4.23)	0.47	1.60 (0.60–4.25)	0.50	1.64 (0.62–4.35)
	History of alcohol	No			−0.25	0.78 (0.55–1.09)	−0.25	0.78 (0.56–1.10)	−0.23	0.79 (0.56–1.11)	−0.24	0.79 (0.56–1.11)
	The status of exercise	Yes			0.45	1.57 (1.34–1.84)***	0.46	1.58 (1.34–1.85)***	0.48	1.61 (1.37–1.91)***	0.47	1.60 (1.36–1.89)***
	Health checkup	Yes			2.11	8.27 (7.03–9.72)***	2.13	8.37 (7.12–9.85)***	2.12	8.34 (7.07–9.83)***	2.12	8.33 (7.04–9.85)***
HRQoL	Health profile	High			−0.04	0.96 (0.77–1.19)	−0.06	0.94 (0.75–1.17)	−0.12	0.89 (0.71–1.11)	−0.12	0.89 (0.71–1.11)
	Overall self-rated health status	Medium			0.17	1.19 (0.96–1.47)	0.16	1.17 (0.95–1.45)	0.11	1.12 (0.91–1.39)	0.11	1.11 (0.90–1.38)
		High			0.32	1.38 (1.04–1.82) *	0.29	1.33 (1.01–1.77)*	0.27	1.31 (0.99–1.74)	0.27	1.31 (0.99–1.74)
**Interpersonal network**													
Marital status	Married						0.48	1.62 (1.13–2.34)**	0.42	1.52 (1.05–2.20)*	0.41	1.51 (1.04–2.18)*
Number of children	1 or above						1.15	3.14 (1.50–6.60)**	1.17	3.22 (1.52–6.80)**	1.17	3.23 (1.53–6.84)**
Household size	2						−0.57	0.57 (0.34–0.94)*	−0.56	0.57 (0.35–0.95)*	−0.55	0.58 (0.34–0.96)*
	3 or above						−0.46	0.63 (0.40–1.01)	−0.49	0.62 (0.38–0.99)*	−0.48	0.62 (0.38–0.999)*
**Work and life**													
Income	High								0.004	1.00 (0.84–1.20)	0.01	1.01 (0.85–1.21)
Employment	Retired								0.10	1.11 (0.81–1.51)	0.09	1.10 (0.81–1.50)
	Employed								0.48	1.62 (1.29–2.03)***	0.49	1.64 (1.30–2.05)***
	In-school student								−0.90	0.41 (0.11–1.51)	−0.93	0.40(0.11–1.47)
Education	Junior high school/ senior high school								0.12	1.13 (0.92–1.39)	0.11	1.12 (0.91–1.37)
	Technical school								−0.11	0.90 (0.61–1.33)	−0.12	0.88 (0.60–1.31)
	College or above								0.18	1.19 (0.87–1.63)	0.15	1.16 (0.85–1.59)
Distance from the nearest hospital	≥1 km								−0.02	0.98 (0.83–1.14)	−0.02	0.98 (0.84–1.15)
**Social policies enabling resources**													
Family doctors	Yes										−0.06	0.94 (0.73–1.21)
	No										0.04	1.04 (0.82–1.32)
Health records	Yes										0.12	1.13 (0.92–1.39)
	No										0.15	1.16 (0.85–1.60)
Insurance status	Insured										−0.39	0.68 (0.31–1.47)
**-2 Log likelihood**			4,589.267		3,731.028		3,709.682		3,679.150		3,675.080	
**χ2**			239.367		1,097.606		1,118.952		1,149.484		1,153.554	
**Sig**			0.000		0.000		0.000		0.000		0.000	
**Nagelkerke R** ^**2**^			0.088		0.360		0.366		0.374		0.375	

*p < 0.05, **p < 0.01, ***p < 0.001. BMI, body mass index; HRQoL, health-related quality of life.

### Factors associated with BCS and CCS

To explore more specific and clearer information regarding BCS and CCS, a binary logistic regression analysis was conducted. The factors associated with BCS and CCS are shown in Table 4. Age 35–64, having gestational experiences, exercising, going for a health checkup, having children, and being employed were the common positive factors for both women's BCS and CCS. In addition, women who had a chronic disease and those who had a high level of overall self-rated health status were more likely to undergo BCS. Women who were married and had health records had a higher likelihood of undergoing CCS. However, household size was a barrier not only for women's BCS utilization but also for CCS. Obese women were also less likely to be screened for BCS. The Hosmer-Lemeshow (H-L) test showed a good degree of fit for BCS (*p* = 0.427) and CCS (*p* = 0.147). The area under the receiver operating characteristic curve was 0.816 and 0.807 for BCS and CCS, respectively.

**Table 4:** Binary logistic regression analysis of factors associated with BCS and CCS.

**Variables**			**BCS**		**CCS**	
			**β**	**OR(95%CI)**	**β**	**OR(95%CI)**
**Personal traits**						
Age		35–64	0.92	2.50 (1.93–3.23)***	0.94	2.55 (1.97–3.29)***
Gestational experiences		1	1.36	3.89 (2.34–6.49)***	1.41	4.08 (2.33–7.15)***
		2 or above	1.31	3.70 (2.22–6.18)***	1.41	4.09 (2.34–7.17)***
Chronic disease status		With	0.23	1.26 (1.03–1.55)*	0.02	1.02 (0.83–1.25)
BMI		Underweight	0.10	1.10 (0.77–1.58)	0.33	1.39 (0.97–1.98)
		Overweight	0.01	1.01 (0.84–1.22)	0.04	1.04 (0.87–1.25)
		Obese	−0.37	0.69 (0.49–0.97)*	−0.09	0.92 (0.66–1.28)
**Behavior characteristics**						
Health behaviors	History of smoking	No	0.35	1.42 (0.54–3.78)	0.22	1.25 (0.48–3.28)
	History of alcohol	No	−0.25	0.78 (0.55–1.10)	−0.12	0.89 (0.63–1.25)
	The status of exercise	Yes	0.46	1.58 (1.34–1.87)***	0.44	1.55 (1.31–1.83)***
	Health checkup	Yes	2.16	8.65 (7.29–10.25)***	2.01	7.42 (6.30–8.86)***
HRQoL	Health profile	High	−0.06	0.95(0.76–1.18)	−0.14	0.87 (0.70–1.09)
	Overall self-rated health status	Medium	0.19	1.21 (0.97–1.50)	0.12	1.13 (0.91–1.39)
		High	0.32	1.38 (1.04–1.83)*	0.12	1.13 (0.85–1.50)
**Interpersonal network**						
Marital status		Married	0.33	1.39 (0.95–2.02)	0.59	1.64 (1.12–2.40)*
Number of children		1 or above	1.17	3.21 (1.48–6.97)**	1.60	4.95 (1.87–13.13)**
Household size		2	−0.52	0.59 (0.36–0.99)*	−0.64	0.53 (0.32–0.88)*
		3 or above	−0.46	0.63 (0.39–1.02)	−0.57	0.57 (0.35–0.92)*
**Work and life**						
Income		High	−0.05	0.96 (0.80–1.15)	−0.02	0.98 (0.82–1.17)
Employment		Retired	0.24	1.28 (0.93–1.74)	0.23	1.25 (0.92–1.71)
		Employed	0.56	1.75 (1.39–2.20)***	0.54	1.71 (1.36–2.15)***
		In-school student	−0.70	0.50 (0.13–1.86)	−1.46	0.23 (0.03–1.95)
Education		Junior high school/ senior high school	0.13	1.14 (0.93–1.40)	0.05	1.05 (0.85–1.28)
		Technical school	−0.11	0.90 (0.60–1.34)	−0.25	0.78 (0.53–1.16)
		College or above	0.21	1.23 (0.89–1.69)	0.02	1.02 (0.75–1.40)
Distance from the nearest hospital		≥1 km	−0.02	0.98 (0.84–1.15)	−0.03	0.97 (0.83–1.14)
**Social policies enabling resources**						
Family doctors		Yes	−0.02	0.99 (0.76–1.27)	−0.07	0.93 (0.72–1.20)
		No	0.05	1.05 (0.83–1.34)	−0.08	0.92 (0.73–1.17)
Health records		Yes	0.09	1.10 (0.89–1.35)	0.26	1.30 (1.06–1.59)*
		No	0.09	1.09 (0.80–1.50)	0.06	1.06 (0.77–1.45)
Insurance status		Insured	−0.39	0.68 (0.31–1.50)	−0.47	0.63 (0.28–1.40)
**-2 Log likelihood**			3,621.523		3,647.894	
**χ2**			1,181.897		1,087.922	
**Sig**			0.000		0.000	
**Nagelkerke R** ^**2**^			0.384		0.360	

*p < 0.05, **p < 0.01, ***p < 0.001. BCS, breast cancer screening; CCS, cervical cancer screening; BMI, body mass index; HRQoL, health-related quality of life.

## Discussion

Recently, an increasing number of young women have been discovered to have developed BC and CC. Cancer screening is an effective secondary prevention strategy that plays an important role in women's health. Since the implementation of the policy on free screening in 2009, screening rates have increased. However, there is still a gap according to the Healthy China Initiative of 2019–2030. This is the first study based on an ecological perspective to explore the determination of women's “two cancers” screening utilization using hierarchical multiple logistic regression analysis.

The results of this study indicated that the rates of BCS (44.1%) and CCS (40.9%) in all ages were both relatively low. Only 50.7% and 47.8% of women who were eligible for free screening attended BCS and CCS, respectively. These are far from the 2022–2030 targets (11). Thus, increasing women's enthusiasm for screening is desired. The findings of hierarchical multiple logistic regression analysis showed that the latter model was interpreted more strongly than the former model, from Model 1 to 5, for the screening attendance of “two cancers”. The binary logistic regression analysis showed the enabling factors and risk factors for BCS and CCS utilization. The view that screening is determined by multiple factors related to the physical and social environments (15) was verified once again. Thus, it is necessary to consider both proximal and distal factors. Social resources should match with women's screening needs.

There were differences between the age groups. Age 35–64 years was positively associated with screening attendance compared with age 18–34 years. In recent years, the rural women's upper age limitation of participating in the screening program for the “two cancers” was increased from 59 to 64 years (10). The boost may have been given by the policy. In China, the women with 45–55 years of age are at high risk for BC (22). This may make women more alert for screening. Because the incidence age of women's “two cancers” is gradually getting younger, it may be useful to continue to expand the program for a free screening. Women's gestational experiences were also significantly related to BCS and CCS, which is consistent with a previous study in the Midwestern United States (23). Women who have not been pregnant more easily miss the screening because of the erroneous view that they are at a lower risk of cervical cancer. It was not shocking that the BCS and CCS non-attendance rates of the women who did not have a gestational experience were up to 88.9% and 92.7%. Therefore, advocacy and education regarding reasons for the diseases should be strengthened, especially for these women. A previous study showed that women with at least one chronic health condition were more likely to be screened (24). Our study also showed that women with chronic diseases such as hypertension, diabetes, and any other chronic diseases diagnosed by a doctor were significantly screened, especially by BCS. This may be explained by more communication with the doctor for health prevention when they visit the doctor. Some studies have shown that the number of visits to a doctor is positively related to attendance in mammography screening (25, 26). Doctors and other health staff members should strengthen their guidance of screening. Obesity is a recognized risk factor for the development of breast cancer, and obese women are at increased risk of cervical cancer. There are inconsistent results regarding the relationship between BMI and screening in women. A previous study has shown that underweight, overweight, and obese women were more likely to delay breast examination and Pap smear testing compared to women with normal weight (27). Charkhchi et al., however, did not find a significant relationship between obesity and BCS or CCS rates (28). Our findings showed that obesity was a barrier to BCS, which is consistent with a study in China (29), but was not significantly related to CCS. These results indicate that obese women may lack risk awareness regarding the relationship between obesity and BCS or CCS. Thus, it is necessary to provide risk education to women with obesity.

A previous study reported that physical exercise increased clinical breast examination and mammography by 0.21 times and 0.13 times, respectively (30). Our results also showed that weekly exercise was positively associated with screening attendance. Health attitudes may be the reason for participating in screening (31). Jin et al. (32) indicated that women who underwent regular medical checkups were more likely to be screened. Our findings also showed these results. In addition to health attitudes (31) and communication with healthcare staff (32), a possible reason is that the project for “two-cancer” screening may be included in some health checkups. Thus, it is important to promote women's attitudes. It is key to mobilizing women to participate in health checkups. At the same time, a communication mechanism between medical workers and women needs to be established. Surprisingly, history of smoking and alcohol consumption were not significantly related to screening attendance in this study. The data on the effect of smoking and alcohol consumption on screening were also contradictory in a previous study. Some studies reported a positive association between absence of alcohol and smoking and screening (31, 33), while others revealed that individuals who did not drink or smoke were less likely to be screened (34). In addition, some authors have not found any associations (35, 36). The results of this study also revealed that having a high level of health profile was not significantly related to screening attendance, which is not consistent with a study in Korea (37). We deduced the possible explanation that women with a higher health profile were more likely to ignore their health problems than others. Therefore, it is necessary to improve healthcare awareness. However, women with a high overall self-rated health status were more likely to be screened for BC. This means that women may pay more attention to BC than CC. The data also reported that the proportion of participants in BCS was 5.3% higher than that in CCS for women who had higher scores.

Marital status and family are important components of the interpersonal network. The results showed that being married was significantly associated with screening, especially for CCS. In addition, having children was significantly associated with screening attendance. Leinonen's study also showed that being unmarried and having no children predicted non-adherence to CCS (38). Sex life is an important factor affecting the development of CC. In addition, Ogunwale (39) reported that perceptions of support from male partners played an important role in women's CCS. This may explain why married women are more likely to be screened for CC. A previous study revealed that higher levels of social support networks led to a more positive attitude toward preventive healthcare (40). Kristiansen (41) showed that women living in households of two to four persons were less likely to not undergo mammography screening. However, in this study, larger household size was a barrier to women's BCS and CCS. The screening rates of all the women did not reach 50%, which indicated that support from family was weak and that the role of the family was not fully functional. Therefore, knowledge, risk education, and screening-related advocacy may not only be enhanced for women but may also be emphasized for family members. If possible, connectedness to the neighborhood or society should be established.

The findings showed that women who were employed had a higher likelihood of participation in screening than those who were unemployed or out of work. Charkhchi's study also revealed that being employed significantly increased breast screening adherence (28). The reasons for this result may be that women with work have more access to information and knowledge of screening through communication with colleagues (42), and have more opportunities for physical examinations organized by the unit (43) than women who were unemployed or out of work. Those who were retired faced a situation similar to that of unemployed women. Students may have more access to information, but there is no significant association between in-school students and screening attendance. Thus, on one hand, more publicity channels should be expanded. However, the screening awareness among school students should be improved through health education. This study revealed that there were group differences in women's educational level, but that this was not related to the uptake of screening, which is consistent with Charkhchi's (28) study. Higher income was not significantly related to screening attendance, which is similar to the study of Yan (44). For both high-income and low-income women, there was a higher rate of participants choosing non-attendance in this study. There were no significant differences in distance from the nearest hospital. Additionally, it was not significantly associated with screening attendance. A similar result was reported in You's study (45).

Having family doctors and health records was not significantly associated with screening for the “two cancers.” Fortunately, women with health records are more likely to be screened for CC. However, these women's BCS and CCS non-attendance rates both nearly reached 50%. These results indicate that family doctors and health staff do not currently play a role in increasing adherence to screening recommendations. A previous study also showed that lack of physicians' recommendations was one of the barriers identified and was caused by lack of knowledge and awareness of screening benefits (46). Therefore, to improve the attitude of family doctors and health staff, their knowledge popularization and education about screening should be strengthened. Being insured was not significantly related to screening attendance. Over 50% of the insured women did not undergo BCS or CCS. We speculate that this may be related to income. In total, 55.4 % of the women had lower income. The cost of screening cannot be reimbursed through insurance. Low-income women may not be willing to pay extra for screening even if they are insured. Thus, insurance was not a screening predictor. Previous studies have reported similar results (43).

### Limitation

The results are meaningful for promoting the screening of the two cancers. However, this study has some limitations. First, the samples were all from Jiangsu province, so there may be limitations to the nationwide generalization of the conclusions. Second, marital status, children, and families were included in the interpersonal network, which was not sufficient to some extent. Interpersonal interactions in the society or the community are also important. Thus, support from the society or the community may be added in the future. Third, the three Level 5 variables in the hierarchical multiple logistic regression analysis, which were drawn from the NHSS, were not significantly associated. It is possible that more variables regarding social policies should be measured in further studies.

## Conclusion

This study provides an ecological explanation for why women undergo or choose to abstain from BCS and CCS. As the five variables are entered into the regression model with the simultaneous forced entry method, the explanatory power of the model is increased. Both proximal and distal factors should be considered. The findings are of great significance in improving women's “two-cancer” screening service utilization.

## Data availability statement

The original contributions presented in the study are included in the article/supplementary files, further inquiries can be directed to the corresponding authors. Requests to access the publicly available datasets used in this study should be directed to heyuan@njmu.edu.cn.

## Author contributions

YH and YS: methodology, software, and writing-review and editing. YS, YH, MCh, and WL: data curation. YS and YM: visualization. YH: conceptualization, resources, supervision, project administration, and funding acquisition. MCh and WL: resources, supervision, and project administration. All authors: writing original draft preparation, contributed to the article, and approved the submitted version.

## Funding

This study was supported by the National Natural Science Foundation of China (Grant Nos: 71804074 and 72174092). The funder had no role in the study design, data collection and analysis, decision to publish, or preparation of the manuscript.

## Conflict of interest

The authors declare that the research was conducted in the absence of any commercial or financial relationships that could be construed as a potential conflict of interest.

## Publisher's note

All claims expressed in this article are solely those of the authors and do not necessarily represent those of their affiliated organizations, or those of the publisher, the editors and the reviewers. Any product that may be evaluated in this article, or claim that may be made by its manufacturer, is not guaranteed or endorsed by the publisher.

## References

[1] Latest Global Cancer Data: Cancer Burden Rises to 19.3 Million New Cases and10.0 Million Cancer Deaths (2020). Available online at: https://www.iarc.who.int/wp-content/uploads/2020/12/pr292_E.pdf (accessed July 26, 2021).

[2] Bray F Ferlay J Soerjomataram I Siegel RL Torre LA Jemal A. Global cancer statistics 2018: GLOBOCAN estimates of incidence and mortality worldwide for 36 cancers in 185 countries. CA Cancer J Clin. (2018) 68:394–424. 10.3322/caac.21492 30207593

[3] Zheng RS Zhang SW Zeng HM Wang SM Sun KX Chen R. Cancer incidence and mortality in China, 2016. JNCC. (2022) 2:1–9. 10.1016/j.jncc.2022.02.002 PMC11256658 39035212

[4] Cancer Today. (2021). Available online at: https://gco.iarc.fr/today/data/factsheets/populations/160-china-fact-sheets.pdf (accessed December 27, 2021).

[5] Han L. Overview of strategies for cervical and breast cancer screening. BeijingMed J. (2014) 36:893–4. 10.15932/j.0253-9713.2014.11.00215890840

[6] Tabar L Yen MF Vitak B Chen HH Smith RA Duffy SW. Mammographyservice screening and mortality in breast cancer patients: 20-year follow-up before and after introduction of screening. Lancet. (2003) 361:1405–10. 10.1016/S0140-6736(03)13143-1 12727392

[7] American Cancer Society. Breast Cancer Facts and Figures 2009, 2010. Atlanta: Am Cancer Society, Inc (2009). p. 28.

[8] Landy R Pesola F Castanon A Sasieni P. Impact of cervical screening on cervical cancer mortality: estimation using stage-specific results from a nested case-control study. Br J Cancer. (2016) 115:1140e6. 10.1038/bjc.2016.290 27632376 PMC5117785

[9] The National Health Family Planning Commission of thePeople's Republic of China. Rural Women “Two Cancers” Examination Project Management Plan. Available online at: http://www.nhfpc.gov.cn/zwgkzt/wsbysj/200906/41534.shtml (accessed November 6, 2018).

[10] National Health Commission of the People's Republic of China. Progress of the Two-Cancer Screening Program in Rural Women. (2019). Available online at: http://www.nhc.gov.cn/jkfpwlz/gzdt1ur/201902/6a19776dd4374223a07dfe9f76ed5157.shtml (accessed October 30, 2020).

[11] Government of the People's Republic of China Healthy China Initiative (2019-2030). Available online at: http://www.gov.cn/xinwen/2019-07/15/content_5409694.htm (accessed March 26, 2022).

[12] Gong P. Screening rate of women's cervical and breast cancer and their influential factors in Wenling. Chin J Public Health Manag. (2015) 31:264–265+267. 10.19568/j.cnki.23-1318

[13] Tian T Di J Yang W Wu Q Chen J Wan D. A comparison study on status and influencing factors of knowledge, attitude, and practice related to breast cancer among women in urban and rural areas in 3 provinces of China. Chin J Health Educ. (2018) 34:14–8. 10.16168/j.cnki.issn.1002-9982.2018.01.004

[14] Mao Y Zhu B Jing P He R. Peculiarity, social environmental and in-patient service-based on the perspective of health ecology. J Northwest Univ. (2016) 46:146–58. 10.16152/j.cnki.xdxbsk.2016-02-024

[15] Liang J Qian X Wang L Xu Ai Xu C. Study on determinants of cervical cancer screening service utilization in urban communities in Shanghai, China. Chinese Health Res. (2011) 14:111–3. 10.3969/j.issn.1007-953X.2011.02.018

[16] Brofenbrenner U. The Ecology of Human Development. Cambridge, Massachusetts, Harvard University Press (1979).

[17] Brofenbrenner U. Toward an experimental ecology of human development. Am Psychol. (1977) 32:513–31. 10.1037/0003-066X.32.7.513

[18] Collins AE. Health ecology and environmental management in Mozambique. Health Place. (2002) 8:263–72. 10.1016/S1353-8292(02)00005-9 12399215

[19] Chen CH Kong L. Guidelines for Prevention and Control of Overweight and Obesity in Chinese adults. Beijing: People's Medical Publishing House (2006).

[20] Zhuo L Xu L Ye J Sun S Zhang Y Burstrom K. Time trade-off value set for EQ-5D-3L based on a nationally representative chinese population survey. Value Health. (2018) 21:1330–7. 10.1016/j.jval.2018.04.1370 30442281

[21] Chen ZH Zhang Q Wang Q Feng X. Coverage and associated factors of cervical and breast cancer screening among childbearing women in Jilin province. Chin J Public Health. (2017) 33:1170–3. 10.11847/zgggws2017-33-08-03

[22] Fan L Strasser-Weippl K Li JJ St Louis J Finkelstein DM Yu KD. Breast cancer in China. Lancet Oncol. (2014) 15:e279–89. 10.1016/S1470-2045(13)70567-9 24872111

[23] Greene MZ Hughes TL Sommers MS Hanlon A Meghani SH. Association of pregnancy history and cervical cancer screening in a community sample of sexual minority women. J Womens Health. (2019) 28:526–34. 10.1089/jwh.2018.6960 30118364 PMC6482891

[24] Wang H Gregg A Qiu F Kim J Chen B Wan N. Breast cancer screening for patients of rural accountable care organization clinics: a multi-level analysis of barriers and facilitators. J Community Health. (2018) 43:248–58. 10.1007/s10900-017-0412-x 28861654

[25] Dourado F Carreira H Lunet N. Mammography use for breast cancer screening in Portugal: results from the 2005/2006 national health survey. Eur J Public Health. (2013) 23:386–92. 10.1093/eurpub/cks103 22874736 PMC3662016

[26] Freitas C Tura LF Costa N Duarte J. A population-based breast cancer screening programme: conducting a comprehensive survey to explore adherence determinants. Eur J Cancer Care. (2012) 21:349–59. 10.1111/j.1365-2354.2011.01305.x 22077789

[27] Fontaine KR Heo M Allison DB. Body weight and cancer screening among women. J Womens Health Gend Based Med. (2001) 10:463–70. 10.1089/152460901300233939 11445045

[28] Charkhchi P Schabath MB Carlos RC. Breast, cervical, and colorectal cancer screening adherence: effect of low body mass index in women. J Womens Health. (2020) 29:996–1006. 10.1089/jwh.2019.7739 31928405

[29] Cheng J. Profile and Predictors of Breast Cancer Opportunistic Screening Utilization Among Female in Tianjin Community. [master's thesis]. Tianjin: Tianjin Medical University (2018).

[30] Kirca N Tuzcu A Gözüm S. Breast cancer screening behaviors of first degree relatives of women receiving breast cancer treatment and the affecting factors. Eur J Breast Health. (2018) 14:23–8. 10.5152/ejbh.2017.3272 29322115 PMC5758059

[31] Kriaucioniene V Petkeviciene J. Predictors and trend in attendance for breast cancer screening in lithuania, 2006-2014. Int J Environ Res Public Health. (2019) 16:4535. 10.3390/ijerph16224535 31744058 PMC6887946

[32] Jin SW Lee J Yun Lee H. Analyzing factors associated with decisional stage of adopting breast cancer screening among Korean American women using precaution adoption process model. Ethn Health. (2021) 26:431–47. 10.1080/13557858.2018.1520813 30326735 PMC6531354

[33] Lin SJ. Factors influencing the uptake of screening services for breast and cervical cancer in Taiwan. J R Soc Promot Health. (2008) 128:327–34. 10.1177/1466424007092802 19058475

[34] Sicsic J Franc C. Obstacles to the uptake of breast, cervical, and colorectal cancer screenings: what remains to be achieved by French national programmes? BMC Health Serv Res. (2014) 14:465. 10.1186/1472-6963-14-465 25282370 PMC4282512

[35] Menvielle G Richard JB Ringa V Dray-Spira R Beck F. To what extent is women's economic situation associated with cancer screening uptake when nationwide screening exists? A study of breast and cervical cancer screening in France in 2010. Cancer Causes Control. (2014) 977–83. 10.1007/s10552-014-0397-z 24842393

[36] Ricardo-Rodrigues I Jiménez-García R Hernández-Barrera V Carrasco-Garrido P Jiménez-Trujillo I López de Andrés A. Social disparities in access to breast and cervical cancer screening by women living in. Spain Public Health. (2015) 129:881–8. 10.1016/j.puhe.2015.02.021 25818014

[37] Choi KH Heo J Kim S Jeon YJ Oh M. Factors associated with breast and cervical cancer screening in Korea: data from a national community health survey. Asia Pac J Public Health. (2013) 25:476–86. 10.1177/1010539513506601 24151045

[38] Leinonen MK Campbell S Klungsøyr O Lönnberg S Hansen BT Nygård M. Personal and provider level factors influence participation to cervical cancer screening: a retrospective register-based study of 13 million women in Norway. Prev Med. (2017) 94:31–9. 10.1016/j.ypmed.2016.11.018 27894911

[39] Ogunwale AN Sangi-Haghpeykar H Montealegre J Cui Y Jibaja-Weiss M Anderson ML. Non-utilization of the pap test among women with frequent health system contact. J Immigr Minor Health. (2016)18:1404–12. 10.1007/s10903-015-0287-9 26424729

[40] Abdulah A Leung T. Factors associated with the use of breast and cervical cancer screening services among Chinese women in Hong Kong. PublicHealth. (2000) 115:212–7. 10.1016/S0033-3506(01)00446-2 11429718

[41] Kristiansen M Thorsted BL Krasnik A von Euler-Chelpin M. Participation in mammography screening among migrants and non-migrants in Denmark. Acta Oncol. (2012) 51:28–36. 10.3109/0284186X.2011.626447 22035117

[42] Pan L Li Q Tang W Fei J Ji Y Zhang Y. Analysis of the factors influencing breast cancer early detection behaviors on the rural women of Jiading district in Shanghai by use of the health belief model. Shanghai Med Pharm J. (2013) 60–4. 10.3969/j.issn.1006-1533.2013.16.033

[43] Mu H Yu L Li Y Liu L Zhan X Meng F. Breast and cervical cancer screening and related factors among residents in both urban and rural areas of Liaoning province. Chin J Public Health Manag. (2015) 31:197–198+201. 10.19568/j.cnki.23-1318.2015.02.028

[44] Yan J Wang D Gao J. Attending situation and influential factors of breast cancer check programme among women in rural areas. Chin Health Service Manag. (2017) 34:373-375+387.

[45] You H Gu H Zhang N Fan H Kou Y Cui N. Why hasn't this woman been screened for breast and cervical cancer? evidence from a Chinese population-based study. Public Health. (2019) 168:83–91. 10.1016/j.puhe.2018.12.007 30708199

[46] Ferdous M Goopy S Yang H Rumana N Abedin T Turin TC. Barriers to breast cancer screening among immigrant populations in Canada. J Immigr Minor Health. (2020) 22:410–20. 10.1007/s10903-019-00916-3 31346839

